# A Comprehensive Analysis of Immune Constituents in Blood and Bronchoalveolar Lavage Allows Identification of an Immune Signature of Severe Asthma in Children

**DOI:** 10.3389/fimmu.2021.700521

**Published:** 2021-07-19

**Authors:** Karine Adel-Patient, Marta Grauso, Rola Abou-Taam, Blanche Guillon, Céline Dietrich, François Machavoine, Mélanie Briard, Nicolas Garcelon, Hassan Faour, Antoine Neuraz, Christophe Delacourt, Thierry Jo Molina, Maria Leite-de-Moraes, Guillaume Lezmi

**Affiliations:** ^1^ Université Paris-Saclay, CEA, INRAE, Département Médicaments et Technologies pour la Santé (DMTS), SPI, Laboratoire d’Immuno-Allergie Alimentaire, Gif-sur-Yvette, France; ^2^ AP-HP, Hôpital Necker-Enfants Malades, Service de Pneumologie et Allergologie Pédiatriques, Paris, France; ^3^ Université de Paris, Institut Necker Enfants Malades, Equipe Immunorégulation et Immunopathologie, Inserm UMR1151, CNRS UMR8253, Paris, France; ^4^ Université de Paris, UMRS 1138, INSERM, Sorbonne Paris-Cité, Paris, France; ^5^ AP-HP, Hôpital Necker-Enfants Malades, Service Informatique médicale, Paris, France; ^6^ AP-HP, Centre-Université de Paris, hôpital Necker-Enfant-Malades, Service d’Anatomie et Cytologie Pathologiques, Paris, France

**Keywords:** children, severe asthma, pathogenesis, immune signature, precision medicine

## Abstract

**Background:**

Targeted approaches may not account for the complexity of inflammation involved in children with severe asthma (SA), highlighting the need to consider more global analyses. We aimed to identify sets of immune constituents that distinguish children with SA from disease-control subjects through a comprehensive analysis of cells and immune constituents measured in bronchoalveolar lavage (BAL) and blood.

**Methods:**

Twenty children with SA and 10 age-matched control subjects with chronic respiratory disorders other than asthma were included. Paired blood and BAL samples were collected and analyzed for a large set of cellular (eosinophils, neutrophils, and subsets of lymphocytes and innate lymphoid cells) and soluble (chemokines, cytokines, and total antibodies) immune constituents. First, correlations of all immune constituents between BAL and blood and with demographic and clinical data were assessed (Spearman correlations). Then, all data were modelled using supervised multivariate analyses (partial least squares discriminant analysis, PLS-DA) to identify immune constituents that significantly discriminate between SA and control subjects. Univariate analyses were performed (Mann-Whitney tests) and then PLS-DA and univariate analyses were combined to identify the most discriminative and significant constituents.

**Results:**

Concentrations of soluble immune constituents poorly correlated between BAL and blood. Certain constituents correlated with age or body mass index and, in asthmatics, with clinical symptoms, such as the number of exacerbations in the previous year, asthma control test score, or forced expiratory volume. Multivariate supervised analysis allowed construction of a model capable of distinguishing children with SA from control subjects with 80% specificity and 100% sensitivity. All immune constituents contributed to the model but some, identified by variable-important-in-projection values > 1 and p < 0.1, contributed more strongly, including BAL Th1 and Th2 cells and eosinophilia, CCL26 (Eotaxin 3), IgA and IL-19 concentrations in blood. Blood concentrations of IL-26, CCL13, APRIL, and Pentraxin-3 may also help in the characterization of SA.

**Conclusions:**

The analysis of a large set of immune constituents may allow the identification of a biological immune signature of SA. Such an approach may provide new leads for delineating the pathogenesis of SA in children and identifying new targets for its diagnosis, prediction, and personalized treatment.

## Introduction

Asthma encompasses multiple phenotypes characterized by common symptoms and variable degrees of airflow limitation (1, 2). In most children, asthma is controlled with mild-to-moderate doses of inhaled corticosteroids (ICS). However, approximately 5% of children suffering from severe asthma (SA) remain symptomatic, despite high doses of ICS with other controllers and the control of aggravating factors (3, 4). Children with SA have frequent severe exacerbations, a reduced quality of life, and may account for approximately half of all pediatric asthma-related healthcare costs (5).

Asthma in children has long been considered to be a type 2 (T2) disorder, as it involves Th2 cells, eosinophils, and other innate immune cells, such as mast cells or type 2 innate lymphoid cells (ILC2) (6–9). However, recent studies have shown that bronchoalveolar lavage (BAL) from children with SA may instead display a dominant Th1 signature, with Th17 and Th2 cells in a mixed cytokine milieu and rare ILC2 (10). Other non-T2 cells or cytokines, such as mucosal associated invariant T cells (MAIT), IL-6, IL-9, IL-17, and IL-33, have also been shown to be involved in the pathogenesis of SA (11–15). Recently, we found that children with SA with higher levels of IL-17A secreting MAIT cells (MAIT-17) in BAL experienced more frequent severe exacerbations in the previous year than those with fewer MAIT-17 (16). Moreover, we also recently evidenced that children with SA and frequent exacerbations exhibited a mixed T2/T17 phenotype, whereas those with less frequent exacerbations were characterized by a more pronounced T1 phenotype (17). Overall, this suggests that different asthma phenotypes may result from various pathophysiological mechanisms, i.e. various endotypes, which may involve several cells and markers of T1, T2, and/or T17 inflammation.

To date, few studies have characterized the immune signature of children with SA relative to that of non-asthmatic (NA) children. Most studies on SA have focused on only one type of immune cell or cytokine, with sometimes conflicting results. Although very instructive, such approaches cannot account for the complexity of inflammation in SA. A better understanding of airway and blood inflammation requires expanding beyond the classical paradigms and considering a global approach rather than a targeted one (10, 18). Continuing from our previous work (17), we aimed to identify sets of immune constituents that can distinguish children with SA from age-matched control children using comprehensive, non-targeted, high-dimensional analysis of data on a large set of cytokines and immune cells in blood and BAL.

## Materials and Methods

### Patients

Children with SA, regularly followed in the department of pediatric pulmonology and allergy of Necker Hospital, were recruited as previously described (16). Institutional ethical approval and written informed consent were obtained.

SA was defined, according to guidelines, as persistent despite the use of high-dose ICS and another controller medication (3, 16). Before the diagnosis of SA was made, good adherence and inhalation technique were confirmed by physicians and underlying modifiable factors and environmental factors were controlled. Children who remained symptomatic underwent investigations, including flexible endoscopy with BAL collection to assess airway inflammation and exclude differential diagnoses (19). Children with severe chronic respiratory disorders other than asthma and requiring flexible bronchial endoscopy for clinical purposes were recruited as age-matched disease-control subjects.

### Bronchoalveolar Lavage (BAL)

Flexible endoscopy was performed at least four weeks after an infection or asthma exacerbation. Cytology, bacterial cultures, and immunofluorescence testing for common viruses were performed (19). After centrifugation, BAL supernatants were collected, aliquoted, and stored at -80°C until cytokine and antibody analysis. Cell pellets were maintained in AIM-V^®^ Medium (ThermoFisher Scientific, Waltham, UK) on ice until labelling (see below).

### Blood Collection and PBMC and Plasma Separation

Blood samples were collected the same day as BAL. Peripheral blood mononuclear cells (PBMCs) and plasma were obtained after Histopaque^®^-1077 (Sigma Aldrich, St Louis, USA) separation. PBMCs were maintained in AIM-V^®^ Medium on ice until labelling. No PBMC stimulation was performed to retain a view of the cell frequency and activation at baseline. The plasma was aliquoted and stored at -80°C for antibody and cytokine analysis.

### Flow Cytometry Analysis

Cell counts and viability determinations were performed using a NovoCyte Flow Cytometer (ACEA Biosciences, San Diego, USA) with 7-Aminoactinomycin D (7-AAD, Interchim, Montluçon, France). PBMCs and BAL were centrifuged (400 x *g*, 5 min, 4°C) and suspended in labelling buffer (PBS, 2 mM EDTA, 2% inactivated fetal calf serum) containing FcR Blocking Reagent (Miltenyi Biotec GmbH, Germany). Extracellular and intracellular labelling of T helper (Th) and innate lymphoid cells (ILC) was performed using a pre-optimized antibody panel for which the spill fluorescence compensation was performed using single-stained UltraComp eBeads™ (Invitrogen, Thermo Fisher Scientific). Cell staining was performed using the following antibodies: lineage (lin: anti-human CD3-, CD11c-, CD14-, CD16-, CD19-, CD56-, FcϵRIα-, CD1a-, and CD123-APC-Vio770™), anti-human CD127 (IL-7Rα)-PE-Vio615™, CD4-VioGreen^®^, CD45-PerCP-Vio700™, Tbet-PE, RORγt-APC, and GATA3-FITC from Miltenyi Biotec Gmb; anti-human IL-13-BV711 and IFNγ-BV605 from BD Biosciences; and anti-human IL-22-eFluor 450 from eBiosciences (Affymetrix, USA). The Fixable Yellow Dead Cell Stain kit (Thermo Fisher Scientific) was used to exclude dead cells. Intracellular labelling was performed after fixation/permeabilization using the Foxp3 Staining Buffer Set (Miltenyi Biotec GmbH, Germany), following the manufacturer’s recommendations. Finally, stained cells were suspended in CytoFix (BD Biosciences, Le Pont de Claix, France) and analyzed using a NovoCyte flow cytometer within 24 h. Analyses were performed using FlowJo^®^ (Version 10, ACEA Biosciences, Inc.). Each experiment contained unlabeled samples and samples labeled with isotype control antibodies. Each acquired sample was first gated on FSC^low^-SSC^low^ cells to select the lymphocyte population and doublet/aggregated cells were unselected using a SSC-A x SSC-H plot. Within the singlet cell population, live CD45^+^ cells were selected. Within this selected population, Lin^+^CD4^+^ (identified as Th) and Lin^-^ cells were gated. Lin^-^ cells were further analyzed for CD127 (IL-7Rα) expression, and ILC identified as Lin^-^CD127^+^ populations. Th1, Th2, and Th17 subpopulations and their ILC analogues ILC1, ILC2, and ILC3, were then identified using intracellular Tbet, GATA3, and RORγt expression, respectively. Th and ILC populations were expressed as the percentage of live CD45^+^ cells to allow comparison. The number of cells was too low to perform a relevant analysis of intracellular cytokines within the BAL. Unlabeled, single-stained, and fluorescence-minus-one (FMO) labelled PMBCs, and PBMCs stimulated for 4 h with phorbol 12-myristate 13-acetate/Ionomycin/Brefeldin were used to validate the gating strategy (not shown).

### Cytokine and Antibody Assays in Plasma and BAL

Cytokines were analyzed using xMAP^®^ Luminex technology and the associated apparatus (Bioplex^®^200, Biorad, Marnes-la-Coquette, France). IL-5 and IL-13 were not detectable or below the limit of quantification in most samples in preliminary experiments (not shown). Then, 40 chemokines (Bio-Plex Pro™ Human chemokine assays, 40-plex; BioRad) and 37 inflammation markers (Bio-Plex Pro™ Human Inflammation Panel 1, 37-plex; BioRad) were analyzed in all BAL and plasma samples following the manufacturer’s recommendations. Samples were incubated with the beads for 18 h at +4°C to increase sensitivity. Due to a small level of redundancy between the kits, 73 immune soluble constituents were analyzed for each sample: APRIL/TNFSF13, BAFF/TNFSF13B, sCD30/TNFRSF8, sCD163, Chitinase 3-like 1, CCL21 (6Ckine), CXCL13 (BCA-1), CCL27 (CTACK), CXCL25 (ENA-78), CCL11 (Eotaxin), CCL24 (Eotaxin-2), CCL26 (Eotaxin-3), CX3CL1 (Fractalkine), CXCL6 (GCP-2), GM-CSF, CXCL1 (Gro-α), CXCL2 (Gro-β), CCL1 (I-309), gp130/sIL-6Rβ, sIL-6Rα, IFNα2, IFNβ, IFNγ, IL-1β, IL-2, IL-4, IL-6, IL-8 (CXCL8), IL-10, IL-11, IL-12p40, IL-12p70, IL-16, IL-19, IL-20, IL-22, IL-26, IL-27 (p28), IL-28A (IFN-2λ), IL-29 (IFN-λ1), IL-32, IL-34, IL-35, CXCL10 (IP-10), CXCL11 (I-TAC), CCL2 (MCP-1), CCL8 (MCP-2), CCL7 (MCP-3), CCL13 (MCP4), CCL22 (MDC), MIF, CXCL9 (MIG), CCL3 (MIP-1α), CCL15 (MIP-1δ), CCL20 (MIP-3α), CCL19 (MIP-3β), CCL23 (MPIF-1), CXCL16 (SCYB16), CXCL12 (SDF-1α+β), CCL17 (TARC), CCL25 (TECK), LIGHT/TNFSF14, MMP-1, MMP-2, MMP-3, Osteocalcin, Osteopontin (OPN), Pentraxin-3, sTNF-R1, sTNF-R2, TLSP, TNFα, and TWEAK/TNFSF12. Total IgG and IgA were also analyzed using the Bio-Plex Pro™ Human isotyping panel (BioRad) and total IgE using in-house specific immunoassays (20). Thus, we analyzed 76 immune soluble components both in BAL and plasma for each individual.

### Statistical Analysis

The sample size was opportunistic, depending on definitive diagnoses and parental consent. Patient demographic and clinical characteristic were compared using the Mann-Whitney (MW) test for quantitative variables and the Fisher exact test for qualitative variables.

Cytokine concentrations and flow cytometry data (acquisition and analysis) were determined blindly and the code was broken only for final statistical analysis. The data for cytokines and cells were not normally distributed. We first assessed correlations between immune constituents measured in BAL and plasma and their correlation with demographic data independently of asthmatic status by calculating Spearman correlations. Among children with SA, we also tested for correlations between immune constituents and clinical data. Correlations with p < 0.05 were considered significant and are provided in the results.

We then performed a descriptive analysis (principal component analysis, PCA) of all immune constituents measured to have an overview of the variables and individuals and identify potential outliers. None were identified and all patients and immune constituents were further used for modelling by supervised partial least square-discriminant analysis (PLS-DA), with the asthmatic status (SA *vs* control subjects) as the explicative variable. Successful construction of the models indicates that it is possible to classify the patients based on all measured immune constituents. The robustness of the models is evaluated based on R²X (explained variance) and R²Y (capability of prediction) scores. Such models allow the identification of “discriminant variables”, that is to say, a set of immune constituents that mostly participated in constructing the models and then mostly supported the differences between the patient groups. These constituents are identified based on model-calculated variable-important-in-projection values (VIP, >1). In parallel, we performed pairwise univariate comparisons of each immune constituent using the non-parametric MW test and the corresponding P values were obtained. We did not adjust for multiple testing; instead, as a final step, the measured immune constituents showing a VIP > 1 and a P value < 0.05 were selected to identify the sets of immune constituents that discriminate the most significantly between children with SA and controls. Such an approach has been proposed for the analysis of metabolomics data, i.e., when the number of variables is (far) greater than the number of individuals (21). Corresponding variables were then represented in univariate graphs to visualize the differences between groups (e.g., extent of increase/decrease, inter-individual variability).

Statistical analyses were performed using R (version 3.6.0; Rcmdr and FactoMineR packages) and XLSTAT^®^ (Addinsoft, Paris, France) software. Graphs were plotted using GraphPad Software, LLC (Prism 8, San Diego, CA, USA). The heatmap was produced using R (ggplot2 package).

## Results

### Patient Characteristics

Twenty children with SA and 10 control subjects were included. General characteristics of the children are summarized in **Table 1**. Children with SA used higher doses of ICS and had a higher post-bronchodilator FEV1/FVC and higher blood eosinophil counts than control subjects. Control subjects had severe chronic respiratory disorders, including ciliary dyskinesia (n = 2), viral pulmonary sequelae (n = 3), or non-cystic fibrosis bronchiectasis (n = 5). Although certain control subjects were being treated with ICS at the time of inclusion, the diagnosis of asthma was excluded based on history, response to bronchodilators, spirometry, and nasal nitric oxide testing or tomodensitometry. The general characteristics of the control subjects receiving or not ICS treatment did not differ (not shown). Control subjects had a higher number of neutrophils in their BAL than children with SA. Virology and bacterial analysis of the BAL did not differ between children with SA and the control subjects. Among children with SA, bacterial cultures were positive for five children (3 for *Haemophilus influenzae*, 1 for *Streptococcus pyogenes*, and 1 for *Staphylococcus aureus*) and viruses were found in four children (2 with rhinovirus, 1 with both adenovirus and parainfluenza virus, and 1 with syncytial respiratory virus). In control subjects, bacterial cultures were positive for five children (3 for *Haemophilus influenzae*, 1 for *Haemophilus influenzae* and *Moraxella catarrhalis*, and 1 for *Staphylococcus aureus*) and viruses were found in one child (non SARS-CoV2 coronavirus).

**Table 1:** Demographic and clinical characteristics of the children with SA and the non-asthmatic disease controls (chronic pulmonary inflammation, NA).

Study participants, n	Severe Asthma (SA)	Non-Asthmatic (NA)	p value NA *vs* SA
	20	10	
Age (years)	10.5 (7.7-12.4)	10.7 (7.7-12)	0.82
Gender (female:male)	7:15	4:6	1*
BMI (kg/m²)	17.6 (15.5-23.1)	17.7 (16.2-19.8)	0.98
Atopy history, n	17/20	6/10	0.18*
Post-BD FEV1 (%)	109 (97-116)	92 (86-102; n=9)	0.14
Post-BD FEV1/FVC (%)	87.5 (81.2-90.5)	76 (73-86; n=9)	***0.057***
ACT score	17.5 (13-21.2)	19 (15-21; n=5)	0.78
Number of bursts	5 (2-9)	1 (0-2; n=5)	0.15
ICS (µg/day eq. fluticasone)	500 (500-562.5)	300 (250-500)	**0.007**
Circulating Total IgE (UI/ml)	218 (136.8-671)	43.57 (36.9-281.9)	0.15
Blood leucocytes (x10 ^3^/µl)	6.7 (5.1-7.4)	7.05 (6.3-10.2)	0.24
Blood eosinophils (x10^3^/µl)	0.5 (0.3-0.9)	0.2 (0.17-0.32)	**0.008**
Blood neutrophils (x10^3^/µl)	2.7 (1.9-3.4)	3.8 (2-6.8)	0.21
Blood lymphocytes (x10^3^/µl)	2.5 (1.9-2.9)	2.65 (2.5-2.8)	0.65
BAL eosinophils %	0 (0-0)	0 (0-0)	0.35
BAL neutrophils %	2 (1-4)	38 (15-84)	**0.001**
BAL lymphocytes %	6 (5-8)	4 (3-5)	***0.059***
Positive BAL bacteriology, n	5/20	5/10	0.23*
Positive BAL virology, n	4/20	1/10	0.64*

BMI, body mass index; BD, bronchodilator; FEV1, forced expiratory volume (1 s); FVC, forced vital capacity; ACT, asthma control test. Post-BD FEV, ACT, and the number of bursts were not available for all controls (number indicated within brackets). Median values (and inter-quartile ranges) are provided for quantitative values.

Statistical analysis: groups were compared using the MW test (1,000 Monte Carlo simulations) or Fisher’s exact test (indicated by an *). Bold values are statistically significant (P < 0.05). Trends are indicated by italics.

### Age and BMI Correlate With the Level of Certain Blood and BAL Cytokines

Spearman correlations performed on the entire population showed age to be positively associated with blood IgE concentrations (ρ = 0.387) and negatively associated with blood concentrations of sCD30, IFNα2, IFNγ, IL-2, IL-11, IL-12p40, IL-19, IL-20, IFNλ1, IFNλ2, IL-32, IL-34, IL-35, MMP1, Pentraxin-3, TSLP, CXCL13, CCL11, and CXCL9 (ρ ranging from -0.376 to -0.666). Age was negatively associated with BAL concentrations of sIL-16Rb, IFNγ, IL-6, IL-26, MMP3, sTNFR2, CXCL6, GM-CSF, CCL1, CCL7, CCL20, CXCL1, CX3CL1, and CXCL12 (ρ ranging from -0.372 to -0.498).

BMI was positively associated with blood concentrations of total IgE and APRIL (ρ = 0.474 and ρ = 0.437, respectively) and negatively with sCD30, IFNα2, IL-2, IL-11, IL-20, IL-27p28, IFNλ2, IL-32, IL-34, MMP1, and TSLP (ρ ranging from -0.372 to -0.513). BMI was also negatively associated with BAL concentrations of sCD163, sIL-16Rb, IL-26, IL-34, and TWEAK (ρ ranging from -0.387 to -0.515).

### Correlation of Soluble Immune Constituents Between BAL and Plasma

No correlation between BAL and blood concentrations of any soluble constituents was observed, except for total IgE, total IgA, IL-12p70, MIF, MMP2, TNFα, and TNFSF14, for which the concentrations in BAL and plasma correlated positively.

**Figure 1:**
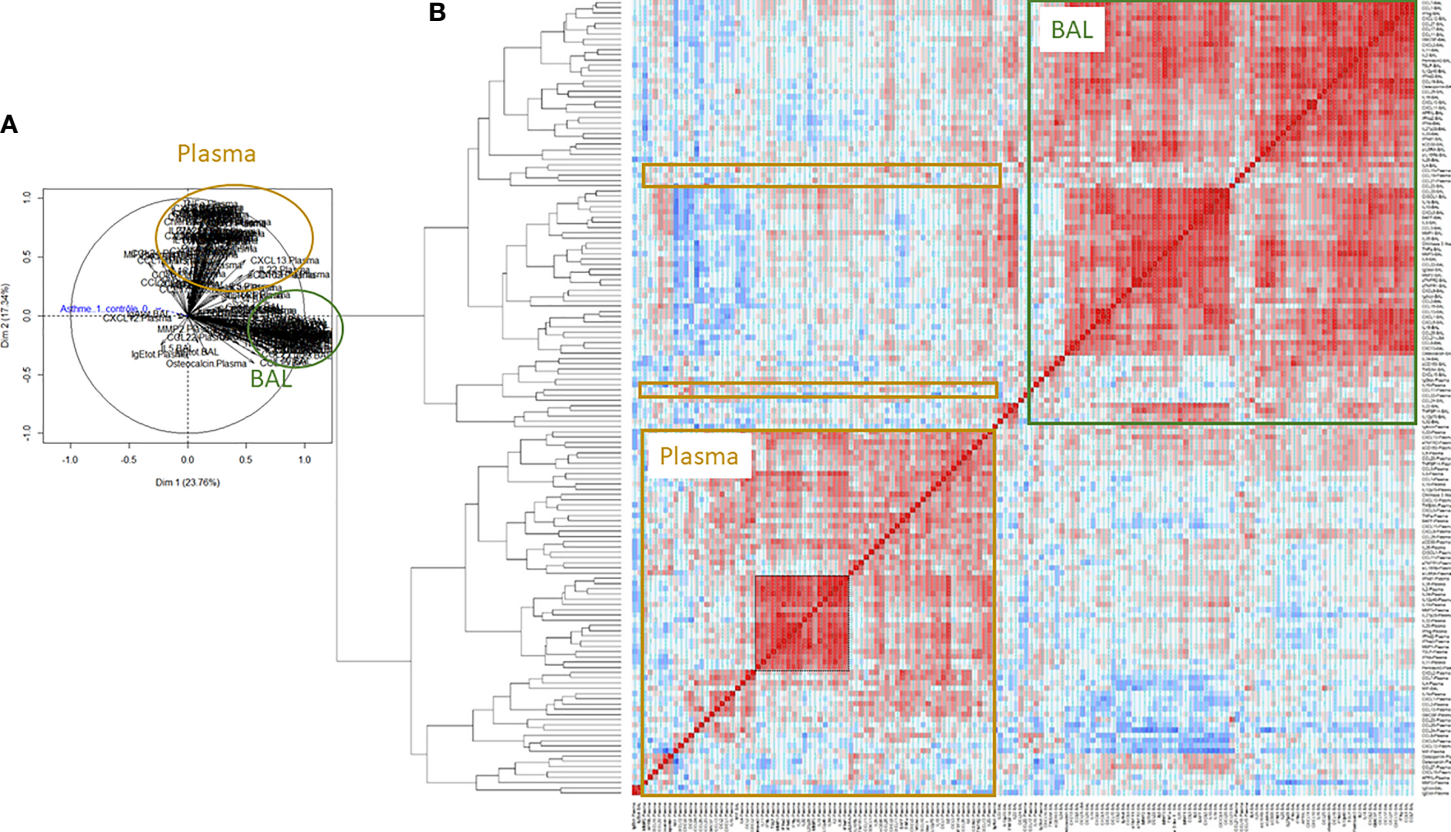
Non-supervised Principal component analysis (PCA, **(A)** and heatmap of Spearman correlations **(B)** for all soluble components analyzed in BAL and plasma from SA and NA (n= 30). Positive correlations are shown in red, the absence of a correlation in white, and negative correlations in blue. The intensity of the color indicates the intensity of the correlation. The black square indicates a core of plasma cytokines that more highly correlated between themselves (comprising IFNα2, IFNβ, IFNγ, IFNλ1, IFNλ2, IL-2, IL-11, IL-12p28, IL-12p40, IL-19, IL-20, IL-32, IL-34, IL-35, TSLP, MMP1, MMP3, and Pentraxin-3).

Moreover, PCA (**Figure 1A**) and a correlation heat map (**Figure 1B**) for all cytokines and antibodies showed only a few correlations between BAL and plasma. Within BAL, most of the constituents positively correlated with each other. Within plasma, a particular core of cytokines was more highly correlated among themselves (comprising IFNα2, IFNβ, IFNγ, IFNλ1, IFNλ2, IL-2, IL-11, IL-12p28, IL-12p40, IL-19, IL-20, IL-32, IL-34, IL-35, TSLP, MMP1, MMP3, and Pentraxin-3, indicated by the black square in **Figure 1B**).

### Correlations of Blood and BAL Immune Constituents With Symptoms of SA Children

Among children with SA, blood concentrations of Pentraxin-3 negatively correlated with the ICS dose (ρ= 0.502) and the blood TSLP concentration correlated positively with the number of crises in the previous year (ρ = 0.450). Blood concentrations of sIL16RA (ρ = -0.530), sTNFR1 (ρ = -0.490), and CXCL16 (ρ = -0.529) negatively correlated with the ACT score. Various BAL constituents (IgG, BAFF, IL-2, IL-8, IL-12p40, osteocalcin, Pentraxin-3, CXC13, CXCL2, CCL1, CCL3, CCL22, CCL26, and GM-CSF) negatively correlated with post-BD FEV1/FVC, whereas blood concentrations of CCL8 and CXCL6 positively correlated with post-BD FEV1/FVC.

Immune cells analyzed in the BAL or blood did not correlate with ICS dose, the number of crises, or ACT score. Conversely, ILC counts and the frequency of ILC2 cells in BAL positively correlated with post-BD FEV1 (ρ = 0.669 and ρ = 0.669, respectively) and the frequency of ILC3 and Th1-IFNγ^+^ cells in blood negatively correlated with post-BD FEV1/FVC (ρ = -0.685 and ρ = -0.573, respectively).

### A Biological Immune Signature Distinguishes Children With SA From Control Subjects

**Figure 2:**
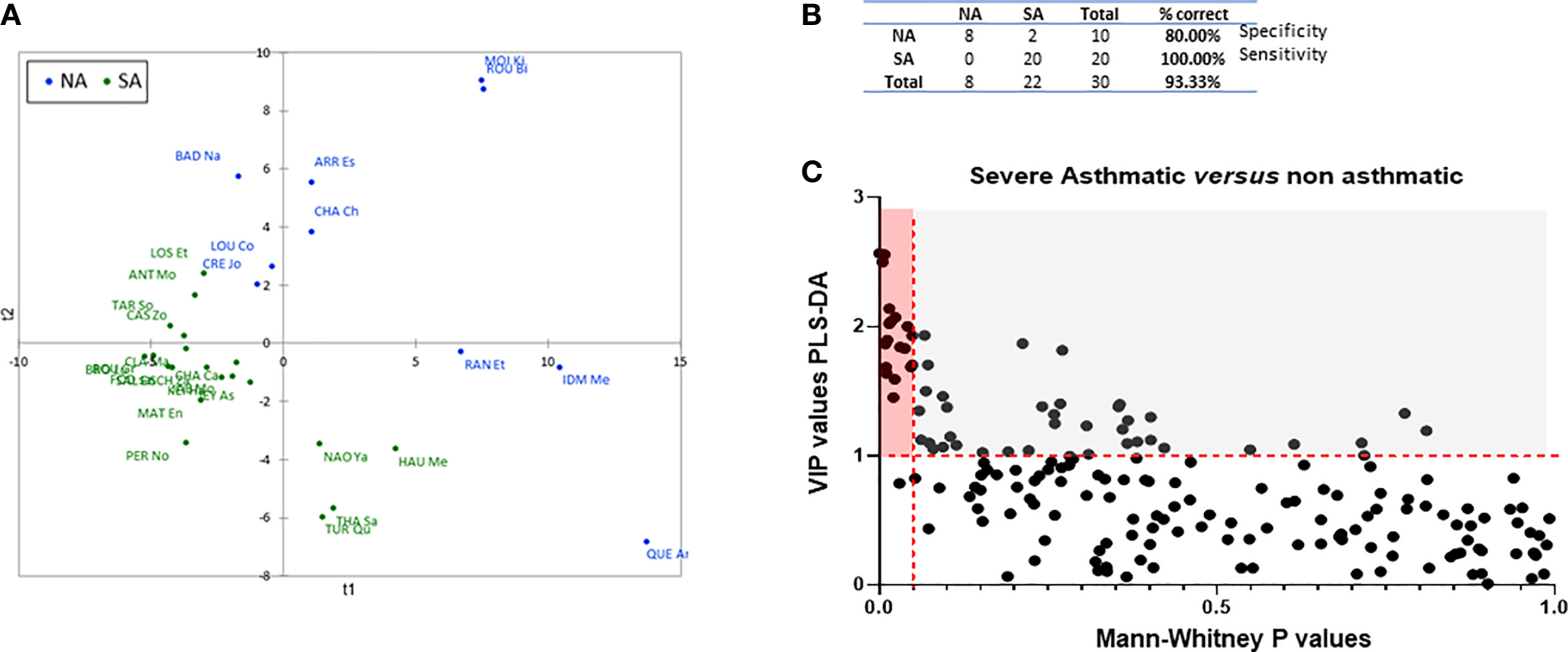
Modelling of all immune constituents measured to discriminate between children with SA and control (NA). **(A)** Graph of all individuals obtained by PLS-DA modelling. SA patients are indicated in green, and NA patients in blue. **(B)** Specificity and sensitivity of the patient classification provided by the PLS-DA modelling. **(C)** VIP x p values plot of all analyzed immune constituents and selection of the most discriminating and significant ones to distinguish between SA and NA patients (red rectangle: VIP > 1, p values < 0.05). The variables that largely participated in the PLS-DA model are shown in the grey region of the graph, i.e., those that belong to the set of variables that allow discrimination between asthmatic and non-asthmatics but show p > 0.05 in the MW test.

We performed a supervised multivariate analysis (PLS-DA) that included all immune constituents (both soluble and cellular) measured in blood and BAL, with asthmatic status as the explanatory variable. Despite the relative heterogeneity of the control subjects, a two-axis model was successfully built (**Figure 2A**), with a good predictive value (R^2^Y = 0.735), 80% specificity, and 100% sensitivity (**Figure 2B**). The VIP values calculated by PLS-DA modelling for all immune constituents are presented in **Table 2**. The variables with a VIP > 1 contributed the most to discriminate between children with SA and controls, and most were measured in BAL.

**Table 2:** VIP values (PLS-DA) and p values (Mann Whitney) for all measured immune constituents for comparisons between children with SA and controls.

Variable	VIP	p value	Variable	VIP	p value	Variable	VIP	p value
**Neutrophils in BAL (%)**	**2.568**	**<0.0001**	ILC2 in BAL (%)	0.976	0.287	CXCL9-BAL	0.509	0.421
**IL8-BAL**	**2.558**	**0.008**	MMP3-BAL	0.952	0.255	CCL25-BAL	0.505	0.654
**BAFF-BAL**	**2.499**	**0.005**	TSLP-BAL	0.950	0.461	CCL27-Plasma	0.492	0.153
**IgGtot-BAL**	**2.138**	**0.015**	IL22-Plasma	0.946	0.155	IgEtot-BAL	0.482	0.945
**sTNFR2-BAL**	**2.072**	**0.024**	CXCL9-Plasma	0.928	0.629	CX3CL1-Plasma	0.482	0.521
**TNFSF14-BAL**	**2.038**	**0.017**	ILC within PBMC (%)	0.927	0.281	ILC2 IL-13^+^ with. PBMC (%)	0.465	0.855
**CCL3-BAL**	**2.022**	**0.015**	IL2-BAL	0.917	0.727	IL1b-Plasma	0.459	0.876
**CCL2-BAL**	**2.001**	**0.042**	IL34-BAL	0.907	0.270	CCL26-BAL	0.451	0.477
IL6-BAL	**1.933**	*0.067*	ILC2 PBMC	0.893	0.250	sTNFR2-Plasma	0.442	0.405
**IL22-BAL**	**1.930**	**0.049**	CCL24-Plasma	0.891	0.160	CCL19-Plasma	0.440	0.574
**CCL20-BAL**	**1.896**	**0.012**	IFNb-Plasma	0.889	0.202	IL8-Plasma	0.436	***0.073***
Neutrophils in blood (nb)	**1.868**	0.212	IL34-Plasma	0.854	0.174	CXCL10-BAL	0.428	0.705
**Eosinophils in blood (nb)**	**1.865**	**0.009**	MMP1-Plasma	0.850	0.151	CCL8-Plasma	0.413	0.442
**IgAtot-Plasma**	**1.841**	**0.031**	CCL1-BAL	0.850	0.324	MMP3-Plasma	0.405	0.964
**CCL22-BAL**	**1.830**	**0.038**	CCL22-Plasma	0.846	0.237	CCL13-BAL	0.395	0.684
Nb of cell in BAL	**1.817**	0.271	IL12p40-BAL	0.826	0.939	CXCL16-BAL	0.387	0.683
**Th1 in BAL (%)**	**1.704**	**0.049**	MIF-BAL	0.825	*0.053*	ILC1 BAL	0.385	0.374
CXCL5-BAL	**1.703**	*0.072*	TNFSF14-Plasma	0.820	0.334	sIL6RA-Plasma	0.382	0.977
**IL10-BAL**	**1.687**	**0.047**	IL11-BAL	0.816	0.811	CCL19-BAL	0.375	0.761
**sTNFR1-BAL**	**1.685**	**0.010**	TSLP-Plasma	0.814	0.362	IL10-Plasma	0.373	0.681
**MMP2-BAL**	**1.636**	**0.011**	sCD163-Plasma	0.813	0.394	ILC in BAL (%)	0.355	0.548
**TNFa-BAL**	**1.593**	**0.023**	CXCL11-Plasma	0.806	0.230	CCL3-Plasma	0.351	0.516
IL1b-BAL	**1.501**	*0.069*	IgGtot-Plasma	0.802	0.399	CCL21-Plasma	0.350	0.685
CX3CL1-BAL	**1.498**	*0.070*	sCD163-BAL	0.801	0.269	CCL23-Plasma	0.346	0.871
IL35-BAL	**1.459**	*0.094*	CXCL6-BAL	0.791	0.438	IL2-Plasma	0.344	0.245
**CCL26-Plasma**	**1.452**	**0.021**	CXCL13-Plasma	0.786	*0.030*	MMP2-Plasma	0.323	0.336
CXCL2-BAL	**1.403**	0.268	IL27p28-Plasma	0.758	0.204	Blood Lymphocytes (nb)	0.318	0.654
CXC13-BAL	**1.399**	0.356	IL35-Plasma	0.757	0.142	Th2 IL13^+^ within PBMC (%)	0.314	0.401
IgAtot-BAL	**1.382**	0.241	IL12p40-Plasma	0.752	*0.089*	CXCL5-Plasma	0.311	0.620
Osteocalcin-BAL	**1.380**	0.354	IL4-Plasma	0.749	0.566	CXCL10-Plasma	0.309	0.988
IL32-BAL	**1.376**	*0.100*	CXCL12-Plasma	0.739	0.658	sIL16Rb-Plasma	0.290	0.728
Lymphocytes in BAL (%)	**1.348**	*0.059*	IgEtot-Plasma	0.734	0.150	IL11-Plasma	0.280	0.888
ILC3 in BAL (%)	**1.329**	0.778	GMCSF-Plasma	0.711	0.742	IFNd1-Plasma	0.266	0.326
Total Leucoc in blood (nb)	**1.321**	0.259	CCL11-BAL	0.693	0.678	IL16-Plasma	0.265	0.892
IL20-BAL	**1.299**	0.402	CCL15-BAL	0.692	0.307	Chitinase3 like1-Plasma	0.248	0.860
CCL7-BAL	**1.273**	0.368	CCL24-BAL	0.685	0.134	CCL27-BAL	0.248	0.969
IL26-BAL	**1.249**	0.260	Eosino in BAL (%)	0.678	0.341	ILC1 within PBMC (%)	0.242	0.943
CXCL12-BAL	**1.232**	0.307	ILC3 within PBMC (%)	0.667	0.223	Th2 within PBMC (%)	0.241	0.853
GMCSF-BAL	**1.204**	0.360	CCL11-Plasma	0.664	0.783	ILC3 IL22^+^ within PBMC (%)	0.230	0.972
CCL23-BAL	**1.194**	0.810	sIL16Rb-BAL	0.658	0.460	sTNFR1-Plasma	0.225	0.760
ILC1 IFNg^+^ within PBMC (%)	**1.149**	0.105	Osteopontin-BAL	0.649	0.615	IL27p28-BAL	0.217	0.846
Chitinase 3 like 1 - BAL	**1.124**	*0.062*	BAFF-Plasma	0.637	0.603	IFNd1-BAL	0.193	0.387
IFNg-BAL	**1.122**	0.402	sCD30-BAL	0.627	0.229	CCL7-Plasma	0.188	0.230
IL12p70-BAL	**1.108**	0.382	IL19-BAL	0.611	0.809	IFNb-BAL	0.181	0.320
CCL21-LBA	**1.100**	0.714	IL4-BAL	0.607	0.437	Th17 PBMC	0.140	0.336
IL19-Plasma	**1.100**	*0.074*	CXCL2-Plasma	0.598	0.952	IFNa2-BAL	0.133	0.406
CCL13-Plasma	**1.095**	0.367	IL12p70-Plasma	0.591	0.146	CXCL11-BAL	0.131	0.814
CCL8-BAL	**1.089**	0.614	Th17 in BAL	0.591	0.871	Th17 IL22^+^ within PBMC (%)	0.131	0.553
IL26-Plasma	**1.082**	0.114	TWEAK-BAL	0.589	0.781	IL20-Plasma	0.130	0.536
Th2 in BAL (%)	**1.067**	*0.094*	TNFa-Plasma	0.587	0.736	IFNd2-Plasma	0.111	0.324
IL16-BAL	**1.060**	0.422	CCL1-Plasma	0.587	0.934	CCL15-Plasma	0.105	0.337
sIL6RA-BAL	**1.053**	***0.080***	CCL25-Plasma	0.551	0.194	sCD30-Plasma	0.103	0.742
Pentraxin3-BAL	**1.048**	0.549	IL6-Plasma	0.544	0.835	Osteopontin-Plasma	0.087	0.892
MMP1-BAL	**1.041**	0.221	CCL20-Plasma	0.544	0.489	CXCL1-BAL	0.085	0.984
APRIL-Plasma	**1.032**	0.191	IL32-Plasma	0.539	0.260	MIF-Plasma	0.085	0.707
Pentraxin3-Plasma	**1.026**	0.153	CXCL6-Plasma	0.536	0.411	Osteocalcin-Plasma	0.081	0.879
Th1 IFNg^+^ within PBMC (%)	**1.012**	0.310	CXCL16-Plasma	0.533	0.723	IFNg-Plasma	0.066	0.190
CCL17-Plasma	**1.003**	0.718	CCL17-BAL	0.520	0.896	APRIL-BAL	0.064	0.366
Th1 within PBMC (%)	0.997	0.282	CXCL1-Plasma	0.514	0.992	TWEAK-Plasma	0.050	0.966
IFNa2-Plasma	0.981	0.381	CCL2-Plasma	0.511	0.376	IFNd2-BAL	0.010	0.901

Immune constituents showing a VIP > 1 and a trend in the MW test (0.05 < p < 0.1, shown in italics), are underlined. Immune constituents are ranked by decreasing VIP values to highlight the most discriminant immune constituents, which were mostly consistent with those that were significant (MW p values).

Immune constituents showing a VIP > 1 and p < 0.05 are shown in bold.

A pairwise univariate analysis was independently performed to compare all immune constituents between children with SA and control subjects (p values provided in **Table 2**). The VIP values for each constituent were plotted as a function of the p value (**Figure 2C**). Although the PLS-DA model was implemented using all data and all were necessary to discriminate between SA and controls with high precision, 19 immune constituents demonstrating a VIP > 1 (discriminant variables) and p < 0.05 (significant difference) were identified as being particularly relevant (constituents within the red rectangle in **Figure 1C** ***;*** the corresponding constituents are indicated in bold in **Table 2**). Eleven additional variables were also considered to be potentially relevant based on both their VIP and p-value (VIP > 1 with 0.05 < p < 0.1, constituents underlined in **Table 2**).

**Figure 3:**
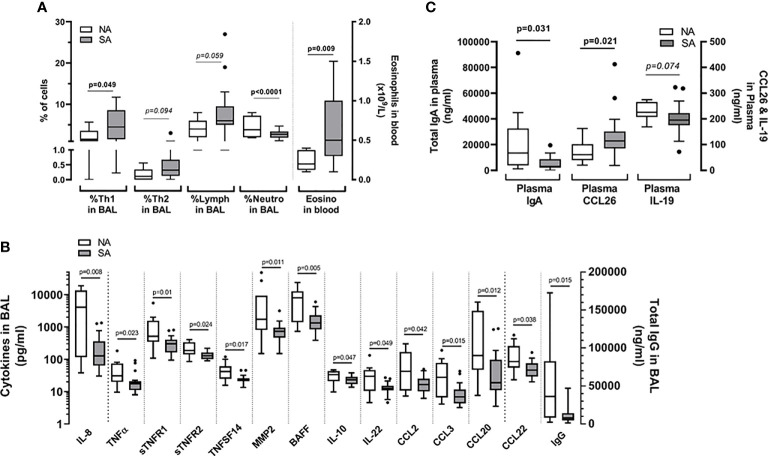
Discriminant immune constituents (VIP > 1) that show significant (p < 0.05) differences or trends towards (0.05 < p < 0.1, indicated in italics) differences between children with SA (grey bars) and NA controls (clear bars). **(A)** Cellular immune constituents. **(B)** Cytokines and IgG concentrations in BAL. **(C)** CCL26, IgA, and IL-19 concentrations in plasma. Exact p values (MW test) are indicated for each constituent.

Statistically significant immune constituents discriminating between SA and NA were then plotted. Children with SA demonstrated higher percentages of Th1 cells in BAL and a higher number of eosinophils in blood than control subjects (**Figure 3A**). The Th2 cell frequency tended to be higher in BAL from children with SA than NA, although the difference was not significant (**Figure 3A**). There were no differences in the percentage of blood or BAL ILCs between children with SA and control subjects (not shown).

In addition to their neutrophilic profile (**Figure 3A**), control subjects had higher levels of total IgG and various inflammatory cytokines (IL-8, TNFα, sTNFR1, sTNFR2, TNFSF14, MMP2, BAFF, IL-10, IL-22, CCL2, CCL3, CCL20, and CCL22) in BAL than children with SA ***(*** **Figure 3B**).

Among the soluble components analyzed in plasma, only two significantly differed between children with SA and the control subjects: IgA, which was lower in children with SA, and CCL26, which was higher in children with SA (**Figure 3C**). We also observed a tendency towards lower concentrations of IL-19 in plasma of children with SA was (**Figure 3C**).

### Preliminary Analysis to Identify Blood Signature of SA in Children

**Figure 4:**
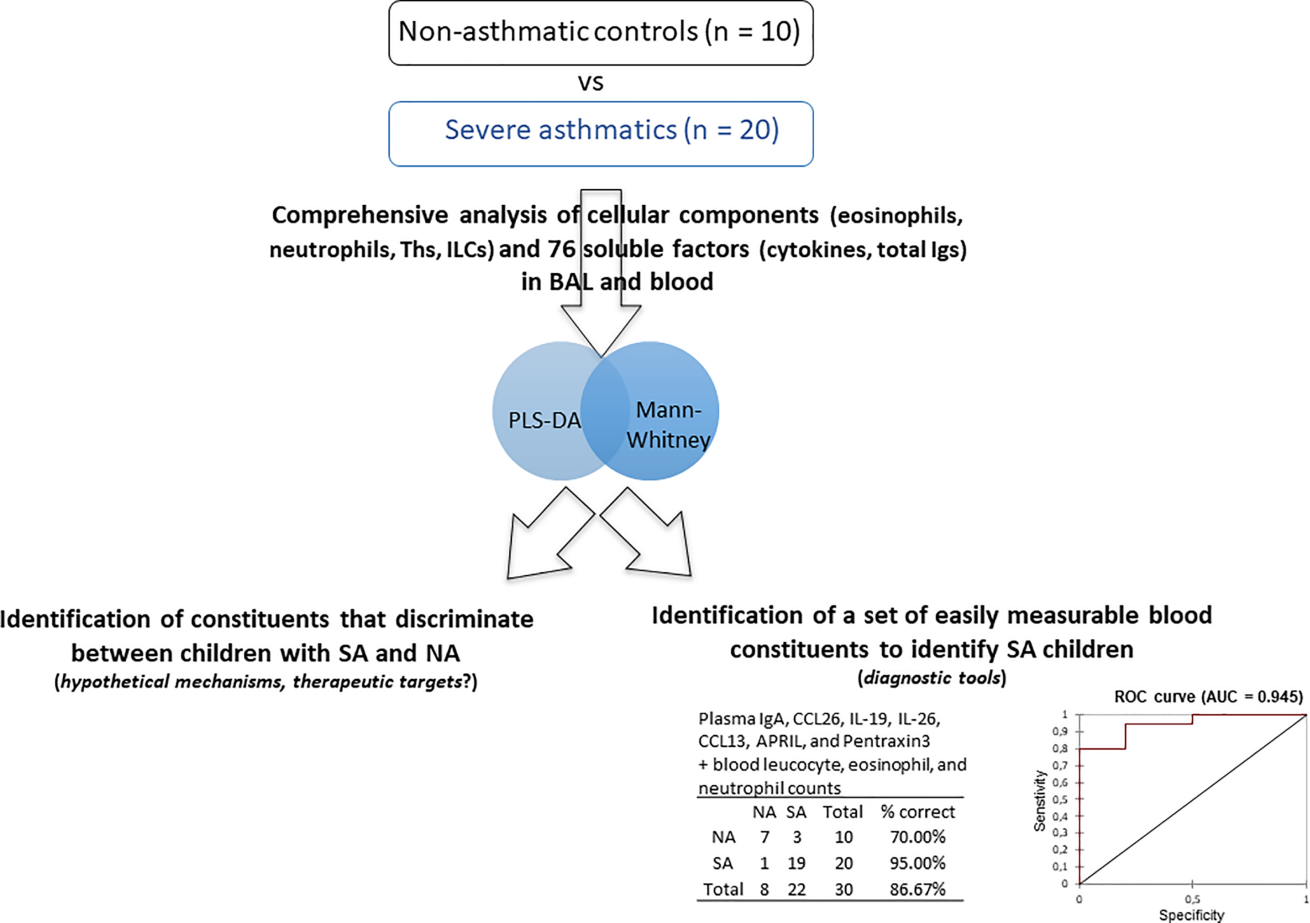
Preliminary analysis to define a set of blood parameters that allow discrimination between children with SA and disease-control children. This model is biased, as the same cohort and parameters used to construct the initial PLS-DA were used to identify the discriminant variables. These results will need to be confirmed on an independent validation cohort.

Although our results were not validated in an independent cohort, we performed preliminary analysis to define a set of “easily measurable” blood variables (to be less invasive than those obtained from BAL) that may distinguish children with SA from NA. We focused on variables measurable in clinical practice in a hospital laboratory and thus excluded data obtained by flow cytometry, which is time-consuming and requires more expertise. Modelling of the most contributive variables identified in blood (IgA, CCL26, IL-19, total leucocytes counts, and number of eosinophils and neutrophils; see **Table 2**) did not provide a good predictive model of the asthmatic status (R²Y = 0.35, classification characteristics: specificity 30%, sensitivity 100%, AUC = 0.68). However, the addition of other blood components with a VIP > 1, i.e., IL-26, CCL13, APRIL, and Pentraxin-3, allowed construction of a model with good predictive value (AUC = 0.945, specificity 70%, and sensitivity 95%) (**Figure 4**).

## Discussion

In this study, a comprehensive and non-targeted analysis of immune constituents both in BAL and blood, combined with high-dimensional analytical approaches, made it possible to distinguish children with SA from control subjects with chronic respiratory disorders other than asthma with relatively high precision. The supervised models were implemented using all measured immune constituents and all constituents were necessary to discriminate between the populations. This reinforces the strength of non-targeted comprehensive analysis to finely characterize clinical phenotypes.

A large set of variables, including cytokines, chemokines, immune cells, and immunoglobulins, were analyzed both in the periphery and in BAL. This point is crucial because i) the levels of many variables in the airways and blood did not correlate with each other in children with SA, as previously demonstrated for eosinophils (22) and Th17 (16) and as shown here when assessing the correlation between blood and BAL cytokines and antibodies, and ii) there may be a substantial overlap between different phenotypes for many of the parameters analyzed (19). It is therefore necessary to multiply the points of view to better characterize the phenotypes of the patients. On the other hand, this confirms that invasive analyses performed in BAL are necessary for a better understanding of the local actors and mechanisms responsible for SA. This is highlighted by the overrepresentation of constituents with a VIP > 1 in BAL.

Endoscopy was performed at least four weeks after any respiratory infection and no children had fever or signs of active respiratory infection at the time of the endoscopy. Despite this precaution, it is a common observation that certain children show positive BAL virology or bacteriology, which may reflect a past infection or chronic colonisation. Among children with SA, those with positive bacteriological cultures had similar counts of BAL neutrophils as those with negative cultures (p = 0.495). They also had similar BAL concentrations of other immune constituents, except for sCD30, chitinase-3-like-1, IL-12p40, IL-26, IFNλ1, IL-35, MMP3, sTNFR1, sTNFR2, and TSLP, which were higher in BAL from children with a positive culture. However, the concentrations of these constituents did not differ between children with SA and control subjects. It is therefore unlikely that the presence of pathogens in BAL affected the analyses.

The analysis of immune constituents that strongly discriminate between children with SA and control subjects shows that children with SA display a mixed Th1/Th2 profile in BAL in a non-neutrophilic environment that is clearly distinguishable from that of control subjects. Our results are consistent with those showing that children with SA display a dominant Th1 signature in BAL, independently of their allergic status (10). In aforementioned study, as in ours, Th17 and Th2 cells were also found in the BAL, whereas ILC2 were scarce. However, we found that the BAL ILC2 frequency correlated with clinical symptoms of children with SA, such as post-BD FEV1, and we cannot exclude an elevated ILC2 frequency in the bronchial mucosa of children with SA.

Conversely, children with SA did not show higher concentrations of cytokines in BAL than control subjects. This confirms the low levels of Th2 cytokines previously observed in BAL from children with severe therapy-resistant asthma (6), although higher concentrations of IL-13 were found in BAL from children with moderate-to-SA vs healthy adults (12). Overall, children with SA showed low levels of all cytokines in BAL, which may result from i) the use of high doses of inhaled steroids; ii) the fact that BAL were collected outside of any asthma exacerbations, and/or iii) a reduced capacity to secrete inflammatory mediators.

Although all data participated in building of the model, we identified the stronger contributors. The fact that certain variables identified to be relevant in our non-targeted analysis were previously identified in other studies using targeted approaches reinforces our results. For example an inverse relationship between plasma IgA concentrations and asthma symptoms has also been shown in adults (23). These observations support possible defective epithelial barrier immunity in children with SA. In addition, the finding that CCL26 strongly contributed to discriminating between children with SA and control subjects is also consistent with data showing that CCL26 is a potent chemoattractant for eosinophils (24, 25) and that elevated concentrations of CCL26 in plasma are related to mucosal counts of eosinophils and the severity of eosinophilic disorders, such as atopic dermatitis (26), chronic rhinosinusitis (27), and eosinophilic esophagitis (28). Moreover, the VIP values and the fact that preliminary modelling integrating the plasma markers IgA, CCL26, IL-19, IL-26, CCL13, APRIL, and Pentraxin-3 allowed good classification of SA versus control children further highlights that these immune constituents may all be useful in characterizing the phenotype of SA. However, this preliminary modelling was biased, as the same cohort and parameters used to construct the initial PLS-DA were used to identify the discriminant variables; these results need to be confirmed using an independent validation cohort.

Our study had several limitations. We are aware that our sample size was small and future studies performed on independent cohorts are required to confirm our results. In addition, age and BMI correlated with certain components. However, age and BMI were comparable between the children with SA and the control subjects. It is therefore unlikely that such associations would have affected our results. The children with SA formed a relatively homogeneous population compared to the non-asthmatic control subjects. The ideal design would have been to recruit healthy children without any respiratory disorders, but this was not feasible for ethical reasons. However, the inclusion of control subjects was critical for identifying the potential distinctive pathological features of SA compared to children suffering from non-asthmatic severe chronic respiratory disorders. The children with SA included here were carefully phenotyped and were all under high doses of ICS. ICS probably affected the levels of both cells and cytokines and thus our results are not transposable to children with non-SA. Furthermore, the biological components were associated with past symptoms and thus the follow-up of the children will be useful to determine the potential predictive interest of the identified immune signatures, or of others not identified in our models.

Our study highlights the complexity of immunological profiles of SA in children and the interest of high-dimensional non-targeted multivariate analysis to provide new leads for delineating the pathogenesis of SA in children and identifying new targets in plasma for diagnosis, prediction, and personalized treatment. Confirming the validity of the models is crucial to determining their pertinence and the potential application of such a global approach in clinical practice.

## Data Availability Statement

The raw data supporting the conclusions of this article will be made available by the authors, without undue reservation.

## Ethics Statement

The studies involving human participants were reviewed and approved by Comité de Protection des Personnes Ile de France 2. Written informed consent to participate in this study was provided by the participants’ legal guardian/next of kin.

## Author Contributions

GL, KA-P, and ML-d-M: designed the research. KA-P, MG, BG, CDi, TM, MB, and FM: performed the research. GL, RA-T, NG, HF, AN, and CD: were responsible for patient recruitment or establishing the patient database. KA-P and GL: analyzed the data. KA-P, GL, and ML-d-M: wrote the manuscript. All authors contributed to the article and approved the submitted version.

## Funding

This work was supported by the INRAE-AlimH Department, a legs Poix grant from the Chancellerie des Universities, Paris, France, and a grant from the ANR (SevAsthma-children, grant no. ANR-18-CE14-0011-01, Paris, France).

## Conflict of Interest

GL reports personal fees from novartis pharma, personal fees from Astra zeneca, personal fees from YSSUP research, during the conduct of the study; personal fees from DBV technologies, personal fees from Aimune therapeutics, outside the submitted work.

The remaining authors declare that the research was conducted in the absence of any commercial or financial relationships that could be construed as a potential conflict of interest.

## Abbreviations

ACT, asthma control test; BAL, bronchoalveolar lavage; BMI, body mass index; FEV1, forced expiratory volume in 1 second; FVC, forced vital capacity; ICS, inhaled corticosteroids; MW, Mann Whitney test; NA, non-asthmatic; PLS-DA, partial least square – discriminant analysis; PCA, principal component analysis; SA, severe asthma; FE, SA with frequent exacerbations; nFE, SA with non-frequent exacerbations.
